# Evaluation of ABT-888 in the amelioration of α-synuclein fibril-induced neurodegeneration

**DOI:** 10.1093/braincomms/fcac042

**Published:** 2022-02-22

**Authors:** Lyndsay Hastings, Arpine Sokratian, Daniel J. Apicco, Christina M. Stanhope, Lindsey Smith, Warren D. Hirst, Andrew B. West, Kaela Kelly

**Affiliations:** 1 Duke Center for Neurodegeneration Research, Department of Pharmacology and Cancer Biology, Duke University, Durham, NC, USA; 2 Biogen Postdoctoral Scientist Program, Biogen, Cambridge, MA, USA; 3 Aiforia Inc., Cambridge, MA, USA; 4 Biogen Neurodegeneration Research Unit, Research and Early Discovery, Biogen, Cambridge, MA, USA

**Keywords:** neurodegeneration, Parkinson’s disease, α-synuclein, veliparib, poly(ADP-ribose) polymerase-1

## Abstract

The accumulation of α-synuclein inclusions in vulnerable neuronal populations pathologically defines Lewy body diseases including Parkinson’s disease. Recent pre-clinical studies suggest poly(ADP-ribose) polymerase-1 activation and the subsequent generation of poly(ADP-ribose) polymer represent key steps in the formation of toxic α-synuclein aggregates and neurodegeneration. Several studies suggest that the inhibition of poly(ADP-ribose) polymerase-1 activity via the poly(ADP-ribose) polymerase-1/2 small molecule inhibitor ABT-888 (Veliparib), a drug in clinical trials for different cancers, may prevent or ameliorate α-synuclein fibril-induced aggregation, inclusion formation and dopaminergic neurodegeneration. Herein, we evaluated the effects of poly(ADP-ribose) polymer on α-synuclein fibrillization *in vitro*, the effects of ABT-888 on the formation of fibril-seeded α-synuclein inclusions in primary mouse cortical neurons and the effects of an in-diet ABT-888 dosage regimen with the intracranial injection of α-synuclein fibrils into the mouse dorsal striatum. We found that poly(ADP-ribose) polymer minimally but significantly increased the rate of spontaneously formed α-synuclein fibrils *in vitro*. Machine-learning algorithms that quantitatively assessed α-synuclein inclusion counts in neurons, both in primary cultures and in the brains of fibril-injected mice, did not reveal differences between ABT-888- and vehicle-treated groups. The in-diet administered ABT-888 molecule demonstrated outstanding brain penetration in mice; however, dopaminergic cell loss in the substantia nigra caused by α-synuclein fibril injections in the striatum was similar between ABT-888- and vehicle-treated groups. α-Synuclein fibril-induced loss of dopaminergic fibres in the dorsal striatum was also similar between ABT-888- and vehicle-treated groups. We conclude that additional pre-clinical evaluation of ABT-888 may be warranted to justify further exploration of ABT-888 for disease modification in Lewy body diseases.

## Teaser

Small molecule poly(ADP-ribose) polymerase inhibitor ABT-888 has been suggested to provide broad neuroprotection in α-synuclein fibril-seeded models of disease. This study evaluated ABT-888 treatment compared with vehicle-only groups but failed to resolve ABT-888 effects in the formation of α-synuclein inclusions or in α-synuclein fibril-induced dopaminergic cell loss in mice.

## Graphical Abstract

**Graphical Abstract:**
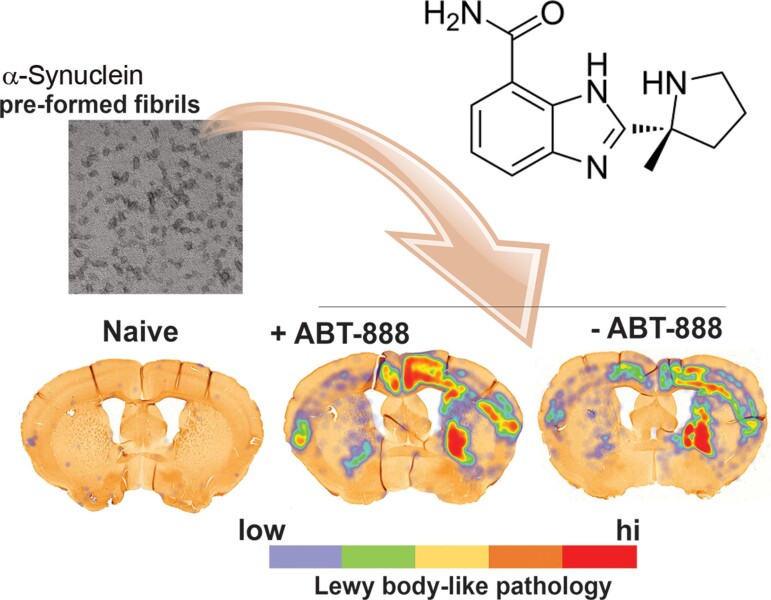

## Introduction

Parkinson’s disease is the second most common neurodegenerative disorder, and most common Lewy body disease, affecting about 1% of people over the age of 60 and totalling over 10 million cases worldwide.^1,2^ Parkinson’s disease is characterized by the loss of the nigral-striatal circuit due to the degeneration of dopaminergic neurons in the substantia nigra pars compacta (SNpc). Lewy bodies, composed in part of fibrillated α-synuclein,^3^ are present throughout the brain, often including much of the frontal cortex in later stages of the disease and in Parkinson’s disease dementia.^4^ Currently, no disease-modifying therapies are available for Parkinson’s disease or related Lewy body diseases, and so there is a strong need for therapeutics development.

Misfolded α-synuclein forms highly structured amyloid fibrils within susceptible neurons that may have the capacity to spread to other vulnerable neurons in a prion-like manner, both in cultured neurons and in the mouse brain.^5–7^ Under pathological conditions, such as through the exogenous exposure of preformed fibrils, soluble α-synuclein undergoes conformational changes and assembles into β-sheet and thioflavin-T positive aggregates.^8^ The surrounding microenvironment within neurons and implicated cofactors might impact the process of α-synuclein fibril formation.^9^

Poly(ADP-ribose) polymerase 1 (PARP-1) and its substrate, poly(ADP-ribose) (PAR), have been broadly implicated in mechanisms relevant to neurodegeneration.^10^ PARP-1 is a nuclear enzyme that, under physiological conditions, is involved in DNA repair by catalyzing PARylation, the addition of ADP-ribose polymers to target proteins. This leads to the recruitment of repair factors and chromatin remodelers.^11,12^ Overactivation of PARP-1 leads to cell death via parthanatos, a cell death pathway that can be triggered by oxidative stress, DNA damage, or nitric oxide production in which PARP-1 overactivation and the subsequent overproduction of PAR causes the release of apoptosis-inducing factor from the mitochondria, ultimately resulting in large-scale DNA cleavage.^11,13^ Dopaminergic neurons in the SNpc have a measured 3-fold higher availability of PARP-1 than other neurons or glial cells,^14,15^ potentially making them more susceptible to parthanatos. In 1-methyl-4-phenyl-1,2,3,6-tetrahydropyridine mouse models of Parkinson’s disease, PARP-1 inhibition and PARP-1/2 knockout improve motor behaviour and prevent dopaminergic neuron loss in the SNpc.^16–18^

Recent studies provide evidence that PAR polymer and α-synuclein colocalize more in the brains of Parkinson’s disease patients than in neurologically normal controls.^19^ Further, evidence *in vitro* and in primary neuronal cultures suggests that PAR polymer formation can both seed and accelerate α-synuclein aggregation.^19,20^ PARP inhibition lessens the accumulation of A53T α-synuclein in transgenic models,^21^ and thus might prevent α-synuclein fibril-induced inclusion formation both in primary neurons and *in vivo* and prevent dopaminergic neurodegeneration.^20^ The PARP-1 isoform knockout has been reported to phenocopy PARP inhibition, so it is thought that PARP-1 is the primary target for the inhibition of PAR polymerization.^20^ For these reasons, PARP-1 inhibitors are attractive molecules for potential disease modification strategies in Lewy body diseases.

Several efficacious PARP inhibitors have been developed and approved for usage in phase clinical trials for the treatment of various cancers.^22^ The drug Veliparib (ABT-888) is one of many known PARP-1/2 inhibitors, but ABT-888 demonstrates evidence of blood–brain barrier penetration in pharmacokinetic (PK) studies, a rare feature for this class of inhibitor.^23^ ABT-888 demonstrates <10 nM binding to PARP-1 and PARP-2,^23^ and drug concentrations tested in the range of ∼100–300 nM have been efficacious in PARP-1/2 inhibition in human blood cells.^24^ Moderate effects have also been described at <10 nM concentrations,^25^ although few studies have attempted to measure unbound ABT-888 concentrations in tissues that might better correspond to earlier *in vitro* binding studies. While currently not approved for the treatment of disease, ABT-888 has currently entered into several Phase III trials with acceptable safety profiles.^26^ In consideration of the pre-clinical evidence, established ABT-888 safety profiles, and favourable brain penetration observed in non-human primates,^27^ ABT-888 thus emerges as a strong candidate for Parkinson’s disease treatment.

It is generally accepted that inter-laboratory replication of key supporting end-points related to the pre-clinical evaluation of clinical candidate therapeutics represents an important aspect of rigour and reliability in contemporary therapeutic discovery efforts.^28,29^ The primary purpose of this study was to replicate the key end-points related to the efficacy of ABT-888 in the amelioration of α-synuclein fibril-induced phenotypes *in vitro* and *in vivo* as principally reported by Kam *et al*.^20^ While our models met the expected variabilities and end-point lesion sizes, α-synuclein inclusion loads were comparable to those previously reported for α-synuclein fibril-seeding-based models,^5,7,30–32^ we were unable to resolve beneficial effects related to ABT-888 treatments. Although we cannot fully reconcile the differences in the present study versus past observations, we speculate underlying factors newly uncovered in this study that may have contributed to the discrepancies.

## Materials and methods

### Production of α-synuclein fibrils and aggregation assays

α-Synuclein fibrils were prepared as previously described.^33^*Escherichia coli* BL21 (DE3) CodonPlus cells (Clontech) with mouse α-synuclein-encoding plasmid (pRK172) were induced with 0.5 mM isopropyl β-d-1-thiogalactopyranoside (IPTG) (RPI) and lysed in 0.75 M NaCl, 10 mM Tris–HCl pH 7.6, 1 mM ethylenediaminetetraacetic acid (EDTA) and 1 mM phenylmethylsulfonyl fluoride (PMSF), with sonication at 70% power (Fisher500 Dismembrator) for 1 min. Boiled lysates (90°C for 15 min) were processed at 20,000 × g for 30 min and filtered supernatants dialyzed to 25 mM NaCl, 10 mM Tris–HCl pH 7.6 and 1 mM PMSF. Equilibrated supernatants were loaded onto a HiPrep Q HP 16/10 Column (Cytiva) for anion-exchange chromatography with the Akta Pure protein purification system (Cytiva), and fractions containing α-synuclein were collected at 300 mM NaCl, Tris–HCl pH 7.6 using gradient elution system. Samples were further dialyzed and concentrated using an ultracentrifugation system with 3K MWCO cutoff columns (Amicon) and remaining endotoxins removed (GenScript) to below 0.1 endotoxin unit per mg level as determined by LAL chromogenic endotoxin quantification (GenScript). The final concentration of the protein was measured by BCA assay (Pierce) and Nanodrop analysis (A_280_). α-Synuclein fibrils were prepared at 7 mg per ml in phosphate-buffered saline (PBS) either in the presence or absence of PAR polymer (concentrations as indicated, Trevigen) at 37°C with continuous orbital shaking. Aggregates were washed and sonicated at 30% amplitude at 10°C in a cup-horn sonicator (QSonica, Q700) for 1 h, with size distributions verified through dynamic light scattering acquisitions (DynaPro) as described.^33^ Aggregation was monitored in real-time under physiological conditions (PBS) supplemented with 10 µM Thioflavin-T (Sigma) on a Fluostar Omega (BMG Labtech) plate reader at 37°C with orbital shaking at 700 rpm for 1 min, followed by 1 min rest cycle. Thioflavin-T fluorescence measurements were acquired every 30 min with time point readings normalized to the maximum intensity of the reaction. All samples were run in triplicates and repeated in three independent experiments on different plates.

### Electron microscopy

Fibril assemblies were prepared at 0.4 mg per ml and added to glow-discharged 300 mesh (Laddreseach industries) stained with 2% uranyl acetate (Electron Microscopy Sciences). Grids were imaged with a FEI Tecnai F20 electron microscope (Eindhoven) operated at 80 kV with nominal magnifications at 30 000× and 66 000× and a defocus range of −1.0 to −1.27 μm. Micrographs were recorded on a Gatan Ultrascan 4000 CCD camera.

### Primary neurons

Mouse cortical neurons (MCNs) were isolated and homogenized from timed-pregnant C57BL/6J mouse embryos (Charles Rivers) on an embryonic Day 17 (E17). Cortices were dissected and washed in Hank’s Buffered Saline Solution and incubated at 37°C with 0.25% Trypsin-EDTA and 1 mg per ml DNase I (Sigma) for 15 min. Cells were resuspended in Neurobasal (Invitrogen) supplemented with 10% foetal bovine serum (FBS, Invitrogen) and 1× GlutaMAX (Invitrogen) and passed through a 100-μm pore cell strainer. For all imaging experiments and plate-based assays, cells were next plated at a density of 20 000 cells per well in black-walled Corning Biocoat Poly-d-Lysine 96-well plates (Thermo). 1.5 h later, media were exchanged for serum-free Neurobasal media supplemented with 2% B27 supplement, 1× GlutaMAX, and 1× Pen/Strep (all from Invitrogen). For immunoblot experiments, MCNs were pretreated with 1 μM ABT-888 for 24 h before adding mouse α-synuclein sonicated fibrils (mPFFs) for 7 days. Cells were collected and lysed on day-*in vitro* (DIV) 14, a time point previously characterized to have elevated PAR levels.^20^ For immunocytochemistry of MCNs, cells were pretreated for 1 h with ABT-888 at the indicated concentration (or vehicle, DMSO) followed by 2 µg per ml mPFFs for 12 days. On DIV 20, cells were fixed in 4% paraformaldehyde and analysed by immunostaining.

### Cell culture treatments

HEK293FT cells (Invitrogen) were cultured in Dulbecco’s Modified Eagle Medium supplemented with 10% FBS, pretreated with DMSO or ABT-888 (2 µM) for 1 h before the addition of N-methyl-N-nitroso-N′-nitroguanidine (MNNG, 20 µM, TCI Chemicals). After 15 min of MNNG exposure, cells were collected into lysis buffer (50 mM Tris pH 7.4, 150 mM NaCl, 1% Triton and 0.1% SDS) supplemented with protease and phosphatase inhibitors (1× complete, Roche). Total protein concentrations were determined by BCA assay as per the manufacturer’s instructions (Pierce). Lysates were diluted into Laemmeli buffer supplemented with 40 mM NaF and fresh 5% dithiothreitol (DTT) for immunoblot analysis. Primary neuron cultures were pretreated with ABT-888 (1 µM) or vehicle (DMSO) for 1 day before the treatment with mPFFs (1 µg per ml) or vehicle (PBS) control. After 7 days, cell cultures were lysed in 1× Laemmeli buffer supplemented with 40 mM NaF and 10% DTT for immunoblot analysis.

### Immunoblots

Cell lysates were electrophoresed on SDS-PAGE 4–20% gradient TGX stain-free gels (BioRad) and transferred to either 0.45 µm Immobilon-FL PVDF membrane (Millipore) or 0.2 µm nitrocellulose membrane (BioRad), followed by immunoblotting with indicated primary antibodies and secondary antibodies: anti-PAR (Trevigen), β-actin-horseradish peroxidase (HRP) (Santa Cruz), goat anti-rabbit HRP (Jackson Immunoresearch) and goat anti-mouse HRP (Jackson Immunoresearch). Chemiluminescent images of membranes were captured digitally using a Chemidoc MP Imaging System Touch (BioRad), with signals developed by Crescendo ECL reagent (Millipore) and quantified with the ImageLab 6.0.1 software (BioRad). Uncropped immunoblot images are shown in Supplemental Fig. 1.

### Immunocytochemistry of dissociated cells

Cells were fixed with 4% paraformaldehyde in PBS for 15 min at room temperature, washed and permeabilized in 0.1% Triton X-100 for 20 min followed by blocking in 0.05% Triton X-100 PBS buffer supplemented with 5% normal goat serum for 1 h. Cells were incubated overnight with primary antibodies as described above in addition to mouse anti-NeuN (clone A60, Millipore) and chicken anti-MAP2 (Abcam). Secondary antibodies included goat anti-mouse IgG, anti-rabbit IgG and/or anti-Chicken IgY conjugated with AlexaFluor fluorescent dyes (Invitrogen). Cells were imaged on a Perkin Elmer Opera Phenix high-content confocal imager at 20× magnification, with images processed electronically with Harmony High-Content Imaging and Analysis (PerkinElmer) and Columbus image analysis platform (PerkinElmer).

### Mice

All animal protocols were approved by local Animal Care and Use Committees accredited by the AAALAC. C57BL/6J non-transgenic mice were obtained from Jackson Laboratories (Stock No: 000664). Approximately equal numbers of male and female mice were used in all experiments, aged 12–14 weeks. All rodents were housed on a 12 h light/dark cycle and given free access to food and water. Consumption of chow, measured as averages per cage, was monitored daily and body weight measured at least thrice weekly. The PARP-1/2 inhibitor Veliparib (ABT-888) was purchased from Selleckchem, purity verified by NMR and TLC at >98% and manufactured into 125 mg per kg rodent chow (NIH 21) by Research Diets Inc. Mice in treatment groups were allowed to acclimate to the chow 7 days before stereotaxic injections, with *ad libitum* access to the chow, which was monitored and replenished as needed. In-diet dosing was continued for the duration of the study (6 months of treatment). Tandem mass spectroscopy was used to analyse concentrations of ABT-888 in the formulated chow as well as in plasma after 7 days of in-diet dosing (*n* = 5), and in plasma and brain 6 months at the end of the study.

### α-Synuclein fibril mouse model and stereotaxic injections

The α-synuclein fibril mouse model consists of C57BL/6J mice, described above, that were injected in the dorsal striatum (dSTR) with preformed α-synuclein fibrils. The mice were assigned to experimental groups using block randomization and controlling for sex to ensure equivalent representation in all study groups. After randomization, mice were anesthetized using isoflurane and unilaterally injected into the dSTR with α-synuclein preformed fibrils (10 µg of protein) over 2 µl of solution in 20 min with a 5 min needle wait before retraction, at the following injection coordinates: 1.0 mm anterior, 1.5 mm lateral to Bregma and 3.0 mm ventral relative to the skull.

### Immunohistochemistry and stereological assessments

Six months post-injection of α-synuclein fibrils, mice were transcardially perfused with PBS and 4% paraformaldehyde, with a 24 h post-fixed in 4% PFA and then 30% sucrose embedding for 24 h at 4°C. All brains were flash frozen in 2-methylbutane and sectioned on a freezing sliding microtome into 40 μm sections. Free-floating coronal sections were processed for tyrosine hydroxylase (TH) staining (AB152, Millipore), or pS129-α-synuclein as described.^31^ Unbiased stereological estimations of the total number of dopaminergic cells in the SNpc were performed using an optical fractionator probe (Stereologer software, Stereology Resource Center) by an investigator blinded to the group identity of the sample. A low-power objective was used to identify the border of the SNpc at all midbrain levels, and sections used for counting covered the entire SNpc and were equally spaced 150 μm apart, with random frame placement through all counting areas. In the context of lesions >50%, the density of the counting frame was increased so that all estimations were based on >100 objects counted per side of the brain. Densitometric quantification of TH signals in the dSTR was performed using far-red shifted (AlexaFluor-800) dyes and image acquisition and the analysis software (Image Lab 6.0.1, BioRad) on a ChemiDoc Touch MP machine (BioRad).

### α-Synuclein inclusion counting

Images with a minimum resolution of 0.24 μm per pixel were uploaded to the Aiforia™ Cloud Version 4.8 image processing and management platform (Aiforia Inc., Cambridge, USA) for processing with deep learning convolutional neural networks (CNNs) and supervised learning. A supervised, multi-layered CNN was trained on annotations from digitized pS129-a-synuclein-stained coronal slices from control sections compared with tissue verified to lack inclusions to count authentic dense-core alpha-synuclein inclusions in single cells.^31,33^ The algorithm was trained on both diverse and representative whole-slide images in the identity-coded dataset (using 25.6% of the total dataset) to create a generalizable machine-learning model. All known artefacts of the staining procedure were identified and trained into the AI model to exclude them from the analysis.^31^

### Statistics and power analysis

Statistical analyses were conducted using the GraphPad Prism 9 software. Specific statistical tests for datasets are outlined in figure legends. Normality was assessed using Shapiro–Wilks and Kolmogorov–Smirnov tests. All tests were performed using significance level of *α* = 0.05 with 95% confidence to determine significant group mean differences (*P < 0.05, **P < 0.01, ***P < 0.001, ****P < 0.0001). Data are presented as mean values with standard error of the mean (SEM). For *in vivo* experiments, a statistical power analysis was performed for sample size estimation based on previous data published for the model,^34^ based on the presumed biological effect size of the treatment,^20^ and based on the effect sizes on the same end-points as observed with antisense oligonucleotides to α-synuclein.^32^ Power and group sizes were centred on the lesion size of the loss of dopaminergic neurons in the ipsilateral side of the injection, where an *α* = 0.05 and power = 0.80 yields a projected sample size of seven capable of resolving 30% effect in the preservation of dopaminergic neurons between treatment and control groups. Samples that did not meet quality control due to tissue staining or sectioning irregularities, abnormalities or suspected perfusion difficulties, were excluded from the final analysis. Sex was evaluated as a covariable in all end-points in this study and did not yield significant differences in mean values between male and female mice, consistent with past studies using this model.^32^

### Data availability

Data that support the findings in this study are available from the corresponding author upon reasonable request.

## Results

### PAR polymer accelerates α-synuclein fibrillization *in vitro*

**Figure 1:**
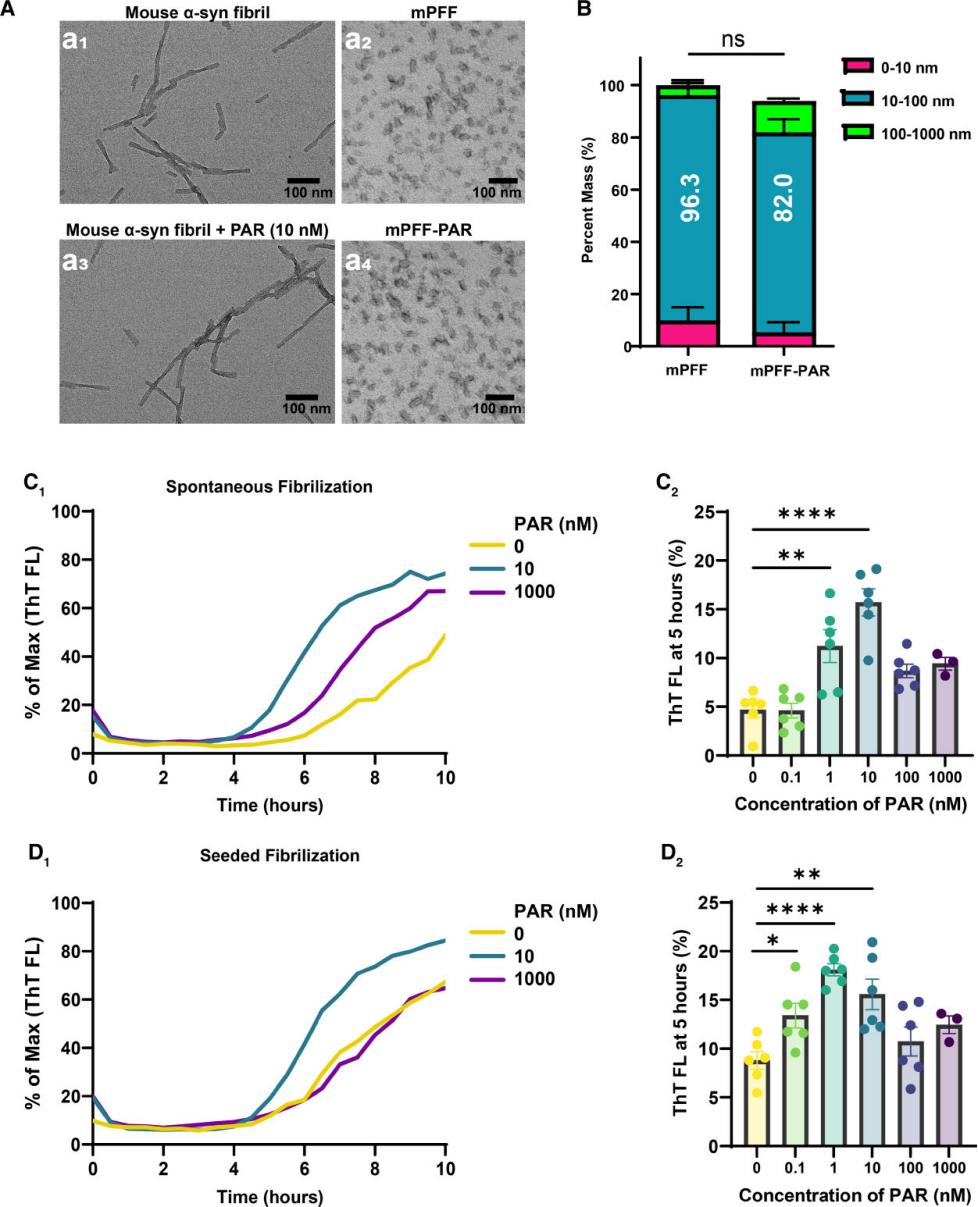
**Accelerated α-synuclein fibrillization with PAR polymer *in vitro*.** (**A**) Representative transmission electron microscopy images of a spontaneously fibrilized mouse α-synuclein before and after sonication (a_2_). Spontaneously fibrilized mouse α-synuclein in the presence of 10 nM PAR before and after sonication (a_4_). The scale bar is 100 nm. (**B**) Dynamic light scattering analysis of sonicated fibrils, with mass distribution binned to 0–10 nm (enriched in monomer protein, shown in magenta), 10–100 nm (enriched in small sonicated fibril particles, shown in blue) and 100–1000 nm (larger fibrils and aggregates, shown in green) [*F*(1,14) = 0.30, *P* = 0.59]. (**C**) Kinetic curves assessing the incorporation of thioflavin-T into spontaneously formed α-synuclein fibrils in the presence of increasing PAR concentrations over time, [*F*(5,27) = 14.6, *P* < 0.0001] and (**D**) assessment of thioflavin-T fluorescence at 5 h past the initiation of elongation [*F*(5,27) = 7.80, *P* = 0.0001]. Each data point represents the measured fluorescence from an independent reaction from an independent well (*N* = 6 per condition) in a single experiment. Data are group means ± SEM. Significance was determined by a two-way ANOVA for main effects in **B**, and one-way ANOVA with a Dunnet’s *post hoc* analysis relative to 0 nM PAR in **C_2_–D_2_**. *P < 0.05, **P < 0.01, ****P < 0.0001.

Preformed α-synuclein fibril models have been widely adopted in pre-clinical research relevant to Parkinson’s disease and related Lewy body diseases.^35^ The molecule ABT-888, known to block the formation of PAR polymer via PARP-1/2 inhibition, has been described to potently ablate the formation of seeded inclusions in neurons and block dopaminergic neurodegeneration caused by intracranial injections of mouse preformed α-synuclein fibrils.^19,20^ One mechanism potentially underlying these observations is that endogenous PAR polymer may critically accelerate the formation of toxic α-synuclein fibrils.^19,20^ To generate the α-synuclein sonicated fibrils (mPFF) particles for investigation in primary neurons and *in vivo* injections, we monitored α-synuclein fibrillization using a real-time quaking-induced conversion assay and observed typical branched fibrillar topology of mouse α-synuclein protein assemblies generated in the presence or absence of 10 nM PAR polymer (Fig. 1A). Sonication of the full-length fibrils resulted in the formation of uniform populations of short α-synuclein preformed fibrils (mPFF, ±10 nM PAR) that showed expected and similar distributions across dynamic light scattering profiles [*F*(1,14) = 0.30, *P* = 0.59] (Fig. 1B). With the titration of PAR polymer into the quaking-conversion assays (PAR chain lengths from 2 to 300 monomers), we observed a biphasic effect not previously described depending on PAR polymer concentration, with an optimal effect on accelerating aggregation at 10 nM [*F*(5,27) = 14.6, *P* < 0.0001] (Fig. 1C). We did not observe a change in lag time for initial spontaneous aggregation at any of the tested concentrations, and at elevated concentrations of 100 nM and 1 µM, PAR polymer lost the acceleration effect [*F*(5,27) = 7.80, *P* = 0.0001] (Fig. 1D). Together, these data support past observations of the 10 nM concentration previously used to accelerate the rate of α-synuclein fibrillization,^19,20^ although the nominal effect appears constrained to the 10 nM concentration.

### Effects of ABT-888 treatment on protein PARylation and pS129-α-synuclein levels and inclusions in primary MCNs

**Figure 2:**
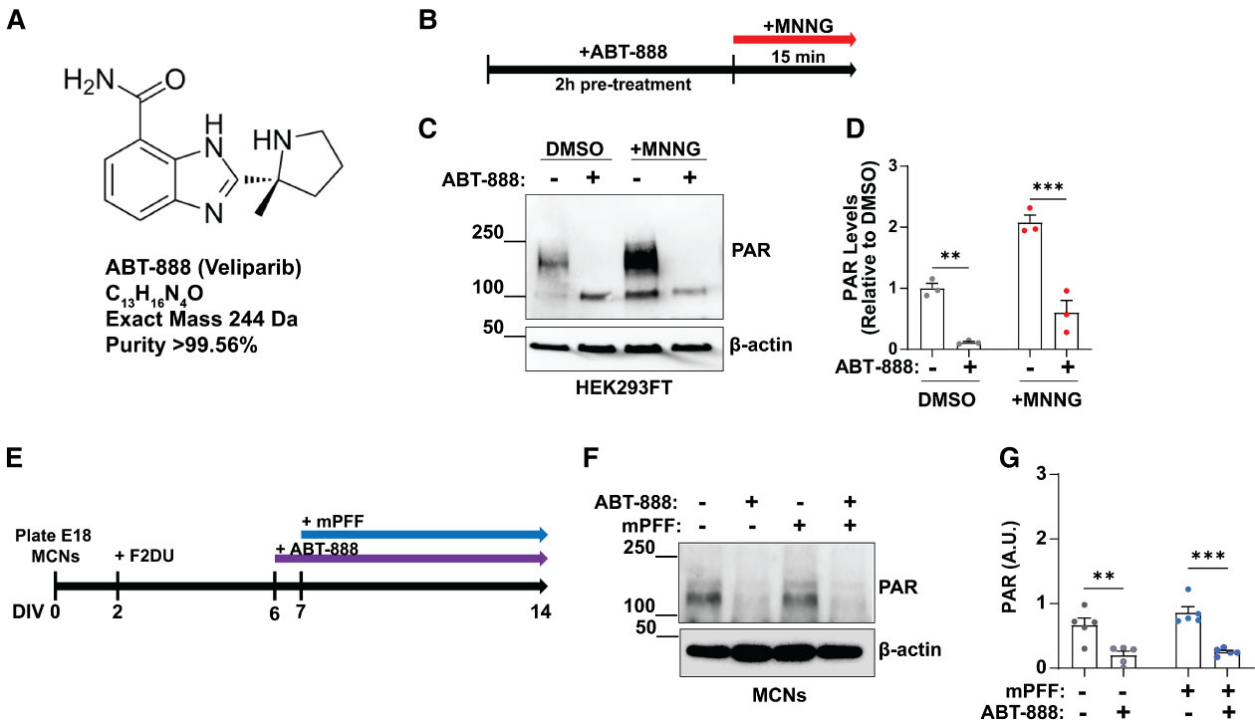
**Effect of ABT-888 on PARylated proteins in cultured cells.** (**A**) Structure and purity of ABT-888 assessed by NMR and (**B**) timeline for ABT-888 treatment on HEK293FT cells in culture with PARP-1/2 activation caused by MNNG. (**C**) Representative immunoblots and (**D**) quantification of PARylated proteins via immunoblot following MNNG (10 μM) or vehicle (DMSO, 0.1%) application in the presence of ABT-888 (1 μM) or vehicle (DMSO, 0.01%) treatment (DMSO: *t*_(4)_ = 10.8, *P* = 0.0004; MNNG: *t*_(4)_ = 6.41, *P* = 0.0031). (**E**) Timeline for ABT-888 treatment of MCNs with or without concurrent treatment of mouse preformed fibrils (mPFFs, 1 μg per ml). (**F**) Representative immunoblots for PARylated proteins and β-actin at DIV 14 neurons, treated with 1 μM ABT-888 or vehicle-only (DMSO, 0.01%) one day before some were exposed to mPFFs (7 days total of fibril treatment). (**G**) Quantification and comparison of possible drug effects on the levels present in total (SDS solubilized and sonicated) protein lysates of high-molecular-weight PARylated proteins normalized to β-actin levels (−mPFF: *t*_(8)_ = 6.40, *P* = 0.0002; +mPFF: *t*_(8)_ = 3.71, *P* = 0.0060). Each data point represents the calculated signal from an independent well of cultured cells. Primary neuron experiment measures were from two different litters of mice cultured at different times. Bar graphs show group means ± SEM. Significance was determined by unpaired two-tailed *t*-tests for evaluating a drug effect, where **P < 0.01, ***P < 0.001. For uncropped immunoblots from **C**, see Supplemental Fig. 2. For uncropped immunoblots from **F**, see Supplemental Fig. 3.

The small molecule ABT-888 (Fig. 2A) has been characterized as a potent (low to sub-nanomolar binding) PARP-1/2 inhibitor with good oral availability and permeability for crossing the blood–brain barrier in tumour models.^23^ To ensure ABT-888 treatment demonstrated the desired effect of limiting PAR polymer formation, we treated HEK293FT cells with MNNG, with or without concurrent ABT-888 (Fig. 2B). MNNG is known to significantly upregulate PAR polymer concentrations in a variety of cell types, including primary cultured neurons.^36–38^ HEK293FT cells treated with MNNG demonstrated a marked increase in PARylated proteins, particularly in the ∼100–250 kDa range, compared with DMSO (Fig. 2C). ABT-888 treatment significantly reduced the concentration of proteins reactive to an anti-PAR antibody as analysed by western blot in lysates from both DMSO- and MNNG-treated HEK293FT cells (*t*_(4)_ = 10.8, *P* = 0.0004; *t*_(4)_ = 6.41, *P* = 0.0031) (Fig. 2D). We then evaluated ABT-888 efficacy for reducing PARylated proteins in primary cultured MCNs, with or without mPFF exposure (Fig. 2E). MCNs pretreated with ABT-888 24 h prior to mPFF treatment demonstrated reduced protein PARylation after 7 days (*t*_(8)_ = 6.40, *P* = 0.0002). Neurons naive to mPFF treatment also exhibited decreases in PARylated protein levels with ABT-888 treatment (*t*_(8)_ = 3.71, *P* = 0.0060) (Fig. 2F and G). Different from past reports, there was no obvious induction of PARylated proteins after 7 days of exposure to mPFFS.^20^

**Figure 3:**
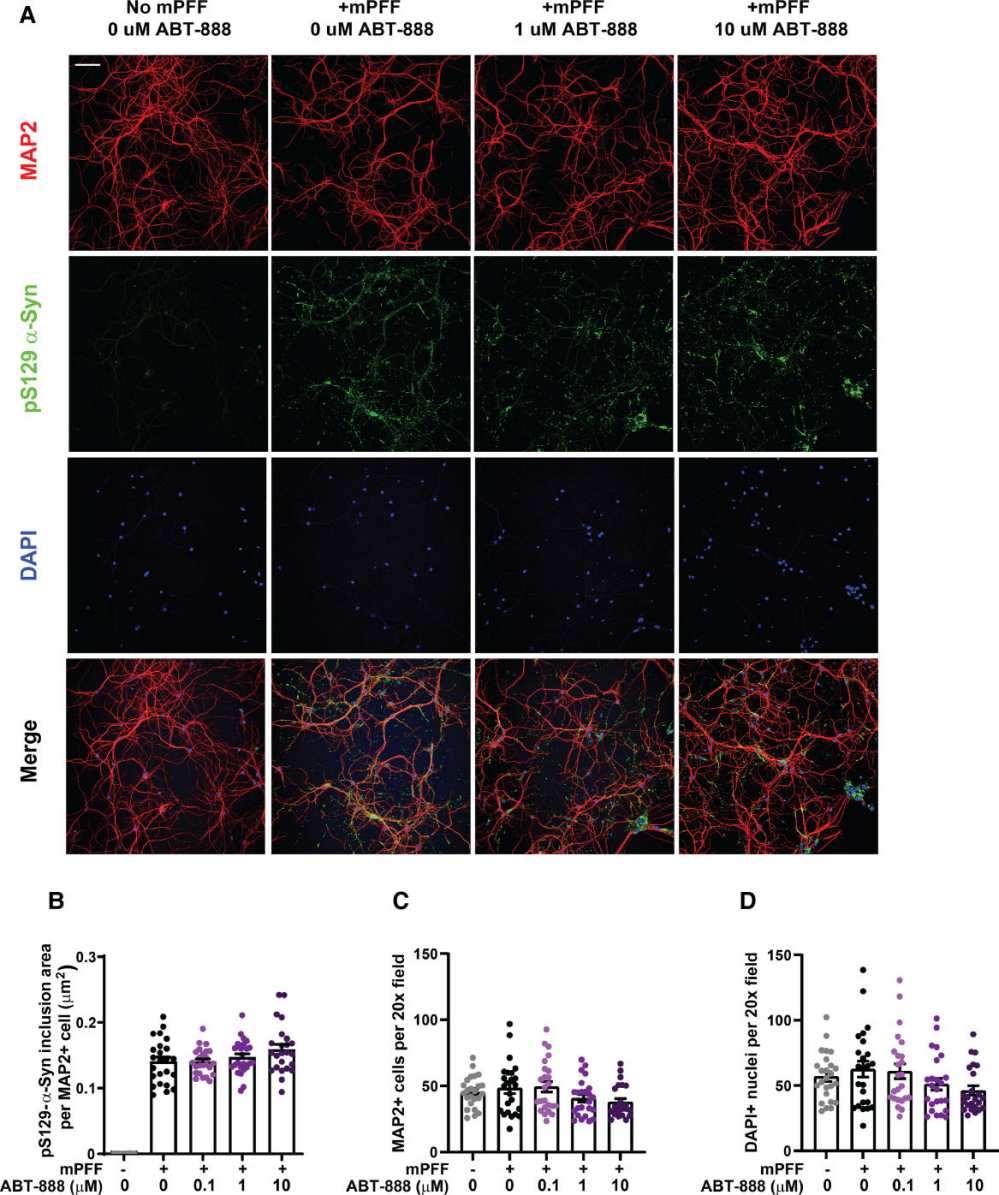
**Effect of ABT-888 on mPFF-seeded pS129-α-synuclein inclusion formation in MCNs.** MCNs were treated with mPFFs (2 μg per ml) concurrently with vehicle-only (0.01% DMSO), or 0.1, 1.0 or 10 μM ABT-888, as indicated, for the duration of the experiment. (**A**) 12-days post-mPFF addition, cells were fixed and high-content confocal images were collected that show MAP2 (red), pS129-α-synuclein (green) and DAPI (blue) signals. Representative images are shown, the scale bar is 0.1 mm. (**B**) Quantification of pS129-α-synuclein inclusion area that overlaps with MAP2+ area (μm^2^) (*H*_(115)_ = 59.85, *P* < 0.0001), (**C**) MAP2+ cell counts measured per 20× field (*H*_(115)_ = 7.78, *P* = 0.1001) and (**D**) DAPI+ nuclei counts per 20× field (*H*_(115)_ = 7.75, *P* = 0.1011). Each data point represents pS129-αsynuclein inclusion area per MAP2 cell area for *N* = 12 wells/group repeated in two independent cultures of primary neurons. Data shown are group means ± SEM with each data point representing the total possible drug effects were evaluated using Kruskal–Wallis tests.

We next sought to determine the effect of ABT-888 on inclusion pathology in the mouse primary cortical neurons, measured with high-content confocal imaging and the pS129-α-synuclein antibody. As expected, a large increase in pS129-α-synuclein reactivity, colocalized with MAP2 signal, was observed with α-synuclein fibril treatment and did not develop in control neurons after 20 days in culture (*H*_(115)_ = 59.85, *P* < 0.0001) (Fig. 3A and B). Meanwhile, the measured pS129-α-synuclein inclusion area per MAP2 cell was similar between vehicle-only-treated wells and ascending concentrations of ABT-888 (0.1, 1.0 and 10 μM) (Fig. 3B). Counts of MAP2 cells and DAPI cells in both fibril treatment and ascending concentrations of ABT-888 were also similar (*H*_(115)_ = 7.78, *P* = 0.1001; *H*_(115)_ = 7.75, *P* = 0.1011), potentially suggesting a lack of cell death caused by either fibril treatment or ABT-888 (Fig. 3C and D). These results suggest that seeded pS129-α-synuclein inclusions may form normally in neurons in the presence of both low and high concentrations of ABT-888.

### Effects of ABT-888 treatment on α-synuclein fibril-induced pS129-α-synuclein inclusions and dopaminergic neurodegeneration in C57BL/6J mice

**Figure 4:**
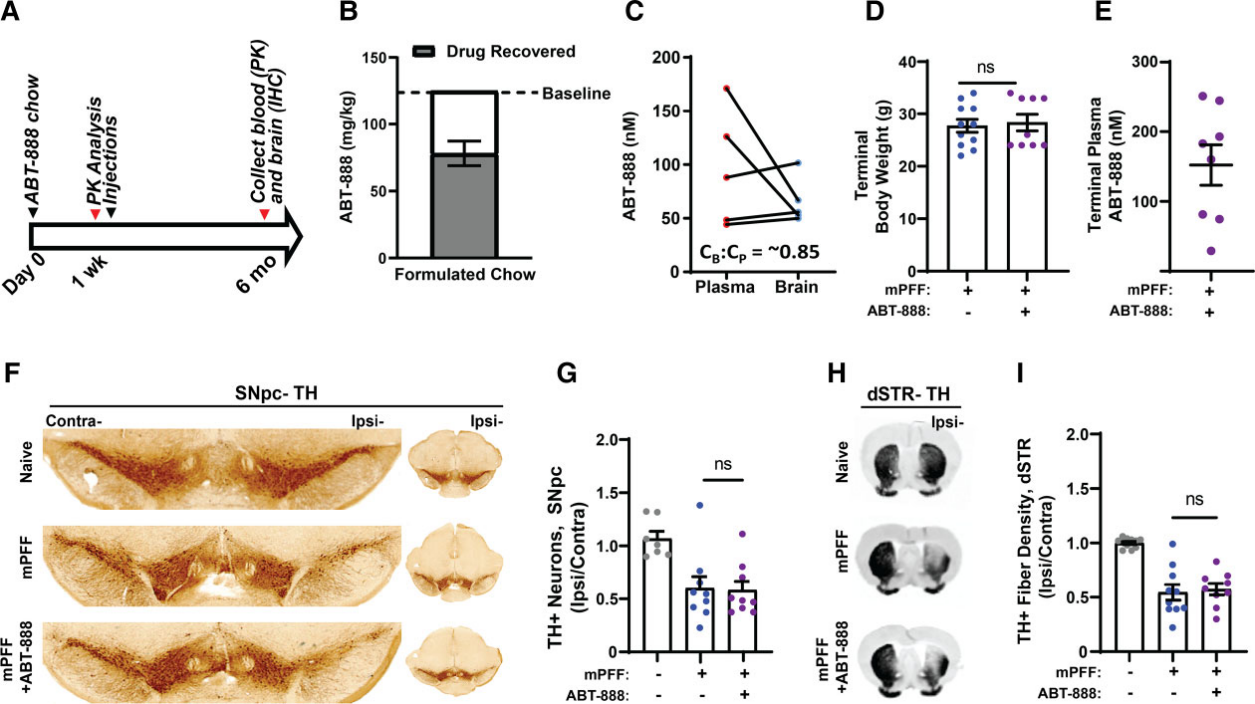
**Effect of ABT-888 on α-synuclein fibril-induced loss of dopaminergic neurons in mice.** (**A**) Timeline describing the ABT-888 in-diet dosing regimen and end-points. (**B**) ABT-888 compound was recovered from the custom-formulated rodent diet. Per cent of the expected recovered drug in comparison to standard curves of drug spiked into control chow homogenates is shown as a bar graph based on three independent mass spectrometry analyses of the common lot of chow used in this study (see ‘Materials and methods’ section). (**C**) As part of the initial PK analysis, ABT-888 drug levels in matched mouse plasma and total brain lysates (PBS-perfused animals) were measured after 1 week of in-diet dosing in five male sentinel mice with the estimated brain to plasma (Cb:Cp) ratio indicated. (**D**) Terminal body weight of mPFF injected mice fed control chow (*N* = 5 females, *N* = 6 males) and ABT-888 formulated chow (*N* = 5 females, *N* = 4 males) (*t*_(18)_ = 0.31, *P* = 0.76). (**E**) Terminal plasma drug concentrations of mPFF injected and ABT-888-treated mice (*N* = 8). One plasma drug measure was not successfully collected. (**F**) Equal numbers of male and female mice (*N* = 7–10 mice per group) were unilaterally injected in the dSTR with 10 μg of mouse preformed fibrils. The animals were randomized, with half the cages treated for the duration of the experiment with ABT-888-medicated chow (mPFF+ ABT-888 group) or control chow (mPFF group). A control cohort (mPFF−, ABT-888−) consisting of naive mouse brain tissue from non-randomized C57BL/6J mice not injected with mPFFs and not treated with ABT-888 were included for reference, but were not included in the evaluation of the possible effects of ABT-888 in order to maximize power. Representative TH immunostaining of SNpc tissue 6 months post-fibril injection. (**G**) Unbiased stereological counts of ipsilateral (mPFF injected side) TH+ neurons, normalized to the measured TH+ neurons on the contralateral side (*t*_(16)_ = 0.09, *P* = 0.9325). (**H**) Representative images of TH+ fibre density in the dSTR 6 months post-mPFF injection and (**I**) quantitative measurements of far-red fluorescence intensity (*t*_(17)_ = 0.32, *P* = 0.7515). Data are graphed as group means ± SEM with each point representing the analysis of an individual mouse. The entirety of the dSTR and SNpc was evaluated in every animal. Possible drug effects were evaluated with unpaired two-tailed *t*-tests between control and ABT-888 chow using log-transformed data to better approximate normal distributions and linearize data. ns, not significant.

To evaluate the possible effects of chronic ABT-888 exposure *in vivo* via an in-diet dosing strategy in the α-synuclein fibril mouse model (Fig. 4A), we first measured brain to plasma ratios of drug from sentinel mice fed medicated chow for 1 week. While PK characterization of ABT-888 has been described in mice, PK parameters and drug distributions are known to dramatically alter with in-diet dosing compared with other delivery approaches like oral gavage,^39^ and to our knowledge, brain concentrations of the ABT-888 molecule have not been measured with in-diet dosing delivery. Rodent diets were formulated to contain 125 mg of ABT-888 per kg of chow, as previously reported.^20^ On average, 62% of unbound ABT-888 compound (equivalent to 78 mg per kg of chow) could be recovered from formulated chow pellets as determined by mass spectrometry analysis (Fig. 4B). In the five sentinel male mice evaluated, a range of ∼50–170 nM of unbound ABT-888 was detected in the plasma, consistent with a previous report of ABT-888 in-diet dosing (100 mg per kg) that also estimated drug plasma levels to be ∼100 nM after 2 weeks of treatment.^40^ We determined unbound ABT-888 in saline perfused brain tissue to harbour an unbound drug brain to plasma ratio (Cb:Cp) of ∼0.85 (Fig. 4C), a higher level of brain penetration than previously reported for the molecule from other formulations in oral and IV delivery strategies.^23^ These results suggest exceptional brain permeability with ABT-888 in-diet dosing in mice. Terminal body weight measurements of mPFF injected mice fed control chow and ABT-888 formulated chow were similar, indicating that drug fed mice were eating normally (*t*_(18)_ = 0.31, *P* = 0.76) (Fig. 4D). Final plasma drug concentrations were variable between mice, but an average 152 nM ABT-888 was detected after 6 months of in-diet dosing (Fig. 4E). Terminal ABT-888 drug levels were only slightly elevated in comparison to the sentinel cohort, suggesting that there was not a drug accumulation effect over time and that proportional brain penetration was achieved.

We next aimed to evaluate whether chronic ABT-888 treatment via in-diet dosing is effective in preventing fibril-seeded α-synuclein inclusions and dopaminergic neurodegeneration, similar to efficacies previously reported.^20^ Six months post-unilateral α-synuclein preformed fibril (mPFF) injection, mice demonstrated an obvious loss of dopaminergic neurons in the SNpc on the ipsilateral side compared with the contralateral side, and compared with age-matched naive controls (Fig. 4F). As assessed through stereological estimations of dopamine-positive neurons in the SNpc, mice injected with mPFFs and fed control chow for the duration of the experiment demonstrated 40.0 ± 11.1% fewer neurons on the ipsilateral side of the dSTR injection compared with the contralateral side. In comparison, mice injected with mPFFs and fed ABT-888-treated chow for the duration of the experiment experienced a 41.7 ± 8.2% loss of dopaminergic neurons in the ipsilateral side compared with the contralateral side (*t*_(16)_ = 0.09, *P* = 0.9325) (Fig. 4G). The lesion size observed in the SNpc was comparable to the proportion of TH fibre loss through the ipsilateral dSTR, which was reduced by 45.5 ± 7.2% in control chow-treated animals and 42.6 ± 5.3% in the ABT-888-treated animals (*t*_(17)_ = 0.32, *P* = 0.7515) (Fig. 4H and I). These results suggest that the loss of dopaminergic neurons was similar between ABT-888 and control chow-treated animals. Drug levels were measured in the plasma and chow at the end of the study and were similar to those concentrations of ABT-888 observed at the beginning of the study.

**Figure 5:**
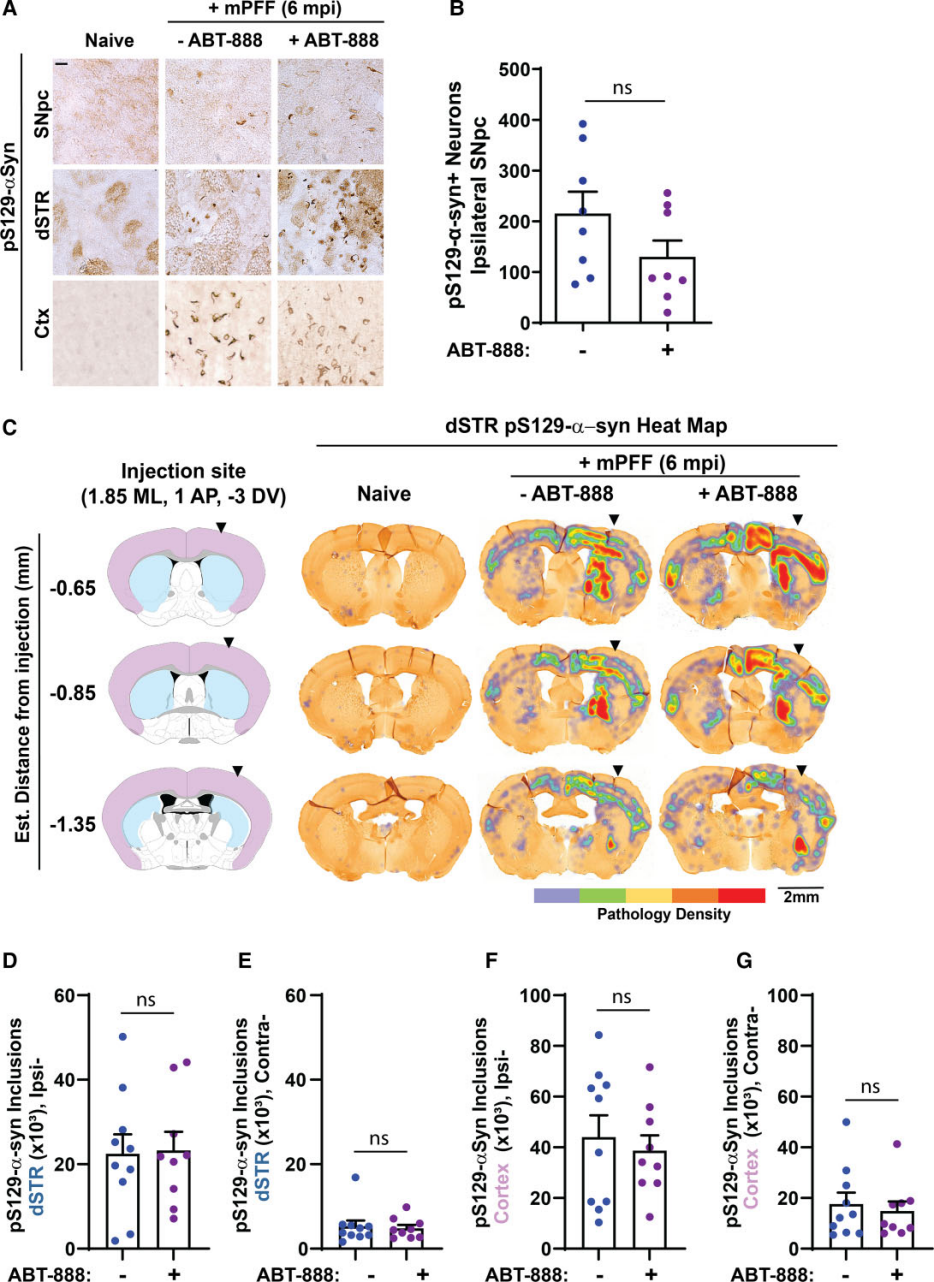
**Effect of ABT-888 on mPFF-seeded pS129-α-synuclein inclusions in mouse dSTR and cortex.** (**A**) Representative images of pS129-α-synuclein immunostaining in the SNpc, dSTR and cortex (Ctx) 6 months unilateral post-injection (6 mpi) of mPFFs into the dSTR. The scale bar is 0.1 mm. (**B**) Unbiased stereological counts of the number of neurons in the substantia nigra bearing pS129-α-synuclein positive inclusions (*t*_(14)_ = 1.64, *P* = 0.1223). (**C**) Integrated heat map representation of pS129-α-synuclein inclusions in sections posterior to the injection site. (**D**) Individual dense-core somatic inclusions were identified throughout the ipsilateral dSTR (*t*_(17)_ = 0.52, *P* = 0.6089), as well as (**E**) inclusions in the contralateral dSTR (*t*_(17)_ = 0.02, *P* = 0.9874) of individual mice, with automated counts performed by custom machine-learning algorithms trained to exclude features present in naive tissues. Likewise, counts through the (**F**) ipsilateral (*t*_(17)_ = 0.04, *P* = 0.9686) and (**G**) contralateral cortex (*t*_(17)_ = 0.32, *P* = 0.7515) were measured. Data are graphed as group means ± SEM with each point representing the analysis of an individual mouse. Sections equally spanning the entirety of the dSTR and the corresponding cortex in the same sections were evaluated in every animal. At least five coronal sections were analysed per animal. Possible drug effects were evaluated with unpaired two-tailed *t*-tests between control and ABT-888 diet using log-transformed data to better approximate normal distributions and linearize data. ns, not significant.

Previously, we found a positive correlation between dopaminergic neurodegeneration and inclusion burden in the mouse α-synuclein fibril model at 6 months post-fibril injection.^30^ To determine whether ABT-888 treatment might alter α-synuclein inclusions 6-months post-unilateral injection of fibrils into the dSTR, we performed unbiased quantitative assessments of individual inclusions through the SNpc, striatum and cortex. The estimated numbers of neurons bearing pS129-α-synuclein inclusions in the SNpc were similar between ABT-888-treated (130 ± 32) and control chow (216 ± 43) groups (*t*_(14)_ = 1.64, *P* = 0.1223) (Fig. 5A and B). Using a high-resolution automated tissue scanning approach, we next developed heat maps that represent local densities of pS129-α-synuclein inclusions across coronal sections that span the dSTR (Fig. 5C). As expected, the density of inclusions was highest near the injection site. We developed a machine-learning algorithm based on rater-curated authentic dense-core inclusions and the exclusion of any pS129-α-synuclein staining features present in naive mice. Automated assessments detected similar numbers of inclusions through the ipsilateral striatum (*t*_(17)_ = 0.52, *P* = 0.6089) (Fig. 5D), as well as the contralateral striatum (*t*_(17)_ = 0.02, *P* = 0.9874) (Fig. 5E) in ABT-888-medicated mice compared with controls. Further, similar numbers of inclusions were recorded in both the ipsilateral cortex (*t*_(17)_ = 0.04, *P* = 0.9686) (Fig. 5F) as well as the contralateral cortex (*t*_(17)_ = 0.32, *P* = 0.7515) (Fig. 5G) in ABT-888-medicated mice compared with controls. Overall, these results suggest that fibril-seeded α-synuclein inclusion burdens and dopaminergic neurodegeneration are similar between ABT-888-medicated animals and controls.

## Discussion

The primary purpose of this study was to evaluate pre-clinical efficacies related to ABT-888 treatment on seeded α-synuclein inclusions and correlated dopaminergic neurodegeneration. The disclosed results are consistent with past reports that ABT-888 effectively reduces the concentration of PARylated proteins, that PAR polymer has the potential to accelerate α-synuclein fibrillization *in vitro* at 10 nM, and that ABT-888 might reduce the amount of pS129-α-synuclein in fibril-treated neurons. However, in both cultured neurons from mice as well as *in vivo*, in the substantia nigra, striatum and cortex, ABT-888 treatment did not have an obvious impact on the number of seeded inclusions. Further, ABT-888 did not noticeably preserve dopaminergic neurons in the substantia nigra in the α-synuclein fibril mouse model compared with the control mice. The apparent lack of efficacy in the prevention or reduction of α-synuclein inclusions or dopaminergic neurodegeneration could not be explained by a lack of ABT-888 penetration in the brain, where the in-diet dosage strategy previously described produced excellent brain penetration of unbound drug concentrations in the brain similar to those in plasma. However, as only a single dose of ABT-888 was used *in vivo* for this study, and target engagement (i.e. PARP-1/2 inhibition) was not established in the brain during disease progression in the model, we cannot rule out the possibility of efficacious ABT-888 doses that might exist at higher concentrations.

PARP inhibition has been demonstrated in numerous studies to provide benefit in a variety of neurodegenerative disease models in mice, from α-synuclein fibril injections,^20^ to TDP-43 toxicity,^41^ and models of Huntington’s disease.^42^ Kam *et al.*^20^ offered a compelling mechanism whereby endogenous PAR polymer accelerates aggregation to facilitate the formation of aggregates with enhanced neurotoxicity. We likewise observed a hastening of fibrilization *in vitro* with 10 nM PAR polymer, but this effect was not accompanied by a decrease in lag time of initial fibrillization, which was consistent with the original report. In contrast, many other agents that include metals and lipids, at physiological concentrations, are known to more dramatically alter the lag time of initial aggregation as well as accelerate fibrillization.^43–45^ If the *in vitro* results for PAR polymer acceleration of fibrillization were extrapolated to neurons in the presence of endogenous levels of PAR polymer, hastened fibrillization after seeded nucleation might result in the expedited appearance of larger, more mature and numerous inclusions. A previous report described peak PAR polymer concentrations to occur after 7 days, although it was unclear why this happened, with further elevations above baseline until at least 14 days.^20^ Interestingly, we did not detect an obvious increase in overall protein PARylation in total lysates after 7 days of mPFF exposure. It is currently unclear how α-synuclein fibrils might induce PARP activation in individual neurons, or whether endogenous PAR levels prior to aggregation facilitate initial seeded nucleations, but further detailed mechanisms may provide critical insights into connecting PAR polymers with aggregated α-synuclein.

We recently demonstrated that manipulating protein interactors of α-synuclein in primary cortical cultures could reduce inclusion formation after α-synuclein fibril treatment.^46^ Investigations into whether ABT-888 inhibition of PARP would similarly reduce neuronal pS129-α-synuclein inclusions was evaluated using the same assays. The observed pS129-α-synuclein surface area occupied within cultured neurons 12 days after fibril exposure was similar between ABT-888-treated cultures (that reduced PARylated proteins) versus control cultures with normal levels of PAR polymer. In possible reconciliation of past reports where ABT-888 nearly ablated the formation of fibril-seeded pS129-α-synuclein inclusions in mouse primary cortical neurons 14 days after fibril treatment,^20^ it seems possible that ABT-888 effects may require elevated PARP activity above endogenous levels. However, the formation of PAR polymer in neurons triggers rapid cell death. Further clarity between the initial triggers of PAR polymer formation, cell death and subsequent inclusion formation (e.g. in other neurons) may provide critical insights into the potential of ABT-888 to intervene in the process.

A recent study demonstrated antisense oligonucleotide treatment targeting α-synuclein mRNA could provide protection from dopaminergic neurodegeneration and the accumulation of α-synuclein inclusions throughout the mouse brain induced by fibril injections in the dSTR.^32^ These studies demonstrate that the same end-points utilized here in a very similar mouse model are indeed modifiable, even when the intervention is applied some weeks after the fibril injections. Our past studies with the α-synuclein fibril model suggest that the inclusion burden in the striatum closely correlates with the observed extent of dopaminergic neurodegeneration in the SNpc.^30^ In this study, we applied advanced image processing approaches to accurately resolve, on a single cell level, the number of distinct dense-core inclusions formed through the substantia nigra, striatum and cortex at 6 months post-injection, a time when there is substantial dopaminergic neurodegeneration. Interestingly, with this approach, inter-variability between mice was similar to that reported by Cole *et al.*^32^ but potentially more variable than comparable measures of inclusion burden and dopaminergic neurodegeneration reported by Kam *et al.*^20^ that used substantially smaller group sizes in some of the experiments (e.g. ABT-888-treated mice assessed from SNpc dopaminergic cell loss). In estimations of study power, with respect to dopaminergic neurodegeneration as assessed by ipsilateral to contralateral cell counts and with the observed 95% CI of 0.34–0.86 in vehicle controls and 95% CI of 0.40–0.77 for ABT-888-treated animals, we would have 80% power to detect a large effect of 42% improvement in lesion size with drug treatment. These results are consistent with observed power resolved in the Cole *et al.*^32^ study. A similar power would be expected in measures of TH-positive fibres in the striatum. As the number of total dopaminergic neurons can vary between mice in biological variability and TH staining in different wells of tissue can cause technical variability between animals, our power further diminishes in the evaluation of raw counts of ipsilateral or contralateral neurons, so we prioritized ipsilateral to contralateral estimations to maximize power. At least in mice in the α-synuclein fibril model, substantial contralateral TH cell loss in the SNpc has not been previously reported, although mild lesioning might have caused a slight underestimation of the ipsilateral lesion that we would not predict to affect our conclusions.

While we cannot conclude that ABT-888 had no effect on inclusion abundance or dopaminergic cell loss, we can conclude that a large effect size in the end-points as previously reported was not observed in this study. Although it might be reasonable to assume that the unbound concentrations of ABT-888 we empirically measured in the brain would be sufficient to inhibit PARP-1/2, future studies might confirm this with better readouts of pharmacodynamic activity than those we were able to establish in this study. Higher drug concentrations may lead to efficacy. However, as it is currently unclear what improvement in SNpc lesioning is required to lead to biologically significant improvement in behaviours, dramatically larger group sizes would be required to resolve smaller effects on lesion size that may have occurred with ABT-888 treatment. Taking our results, together with the recent Cole *et al.*^32^ study using a very similar mouse α-synuclein fibril model used to resolve improvements in inclusion burden and dopaminergic neurodegeneration, we hope that these studies may guide future designs for group sizes and methods of quantification to identify the most promising candidate therapeutics in pre-clinical approaches.

## Supplementary Material

fcac042_Supplementary_Data

## Abbreviations

MCNs =: mouse cortical neurons
MNNG =: N-methyl-N-nitroso-N′-nitroguanidine
PAR =: poly(ADP-ribose)
PARP =: poly(ADP-ribose) polymerase
mPFF =: mouse α-synuclein preformed fibril
SNpc =: substantia nigra pars compacta
