# Supplementary material for: Evaluation of ABT-888 in the amelioration of α-synuclein fibril-induced neurodegeneration
Source: Brain Commun. 2022 Feb 22;4(2):fcac042. doi: 10.1093/braincomms/fcac042 (PMC8907490; doi:10.1093/braincomms/fcac042)
Supplement: fcac042_Supplementary_Data [file fcac042_supplementary_data.docx]

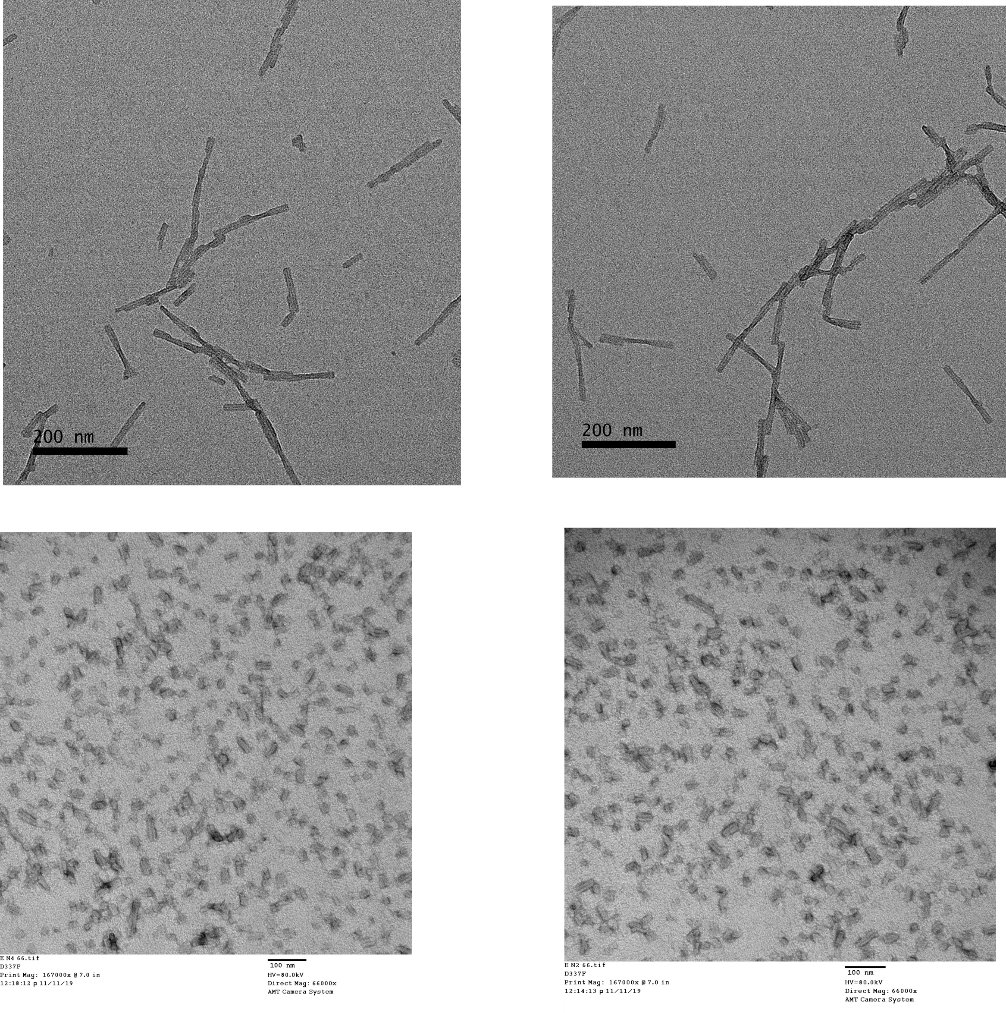


Supplemental Figure 1. Uncropped transmission electron microscopy images from Figure 1A.


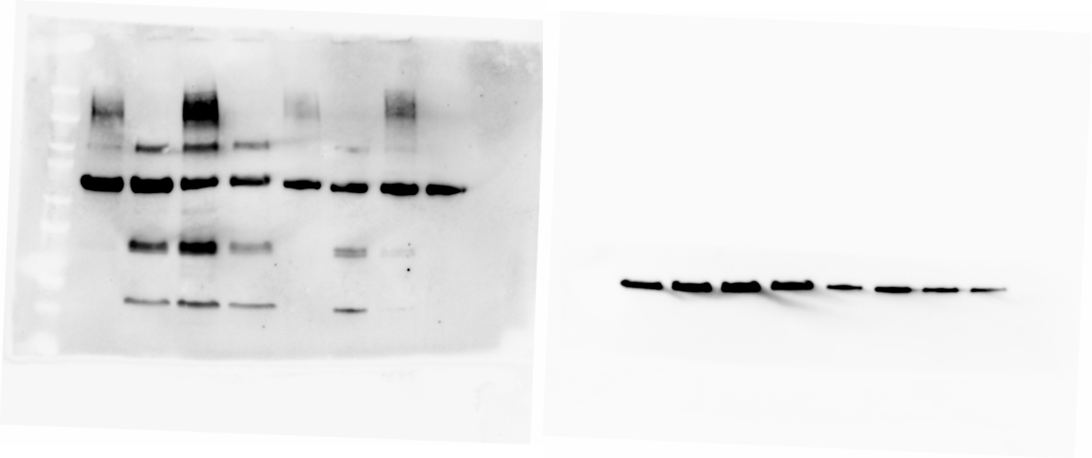


Supplemental Figure 2. Uncropped immunoblots for PAR (left) and B-actin (right) in HEK293FT from Figure 2C. Only the first four lanes were included in the main text.


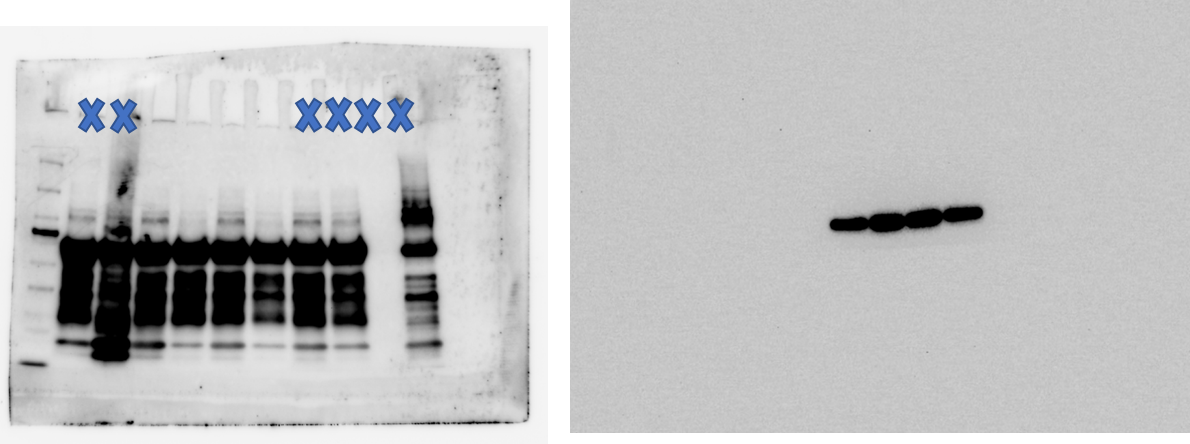


Supplemental Figure 3. Uncropped immunoblots of PAR (left) and B-actin (right) in primary mouse cortical neurons from Figure 2F. Blue X’s indicate lanes that were not included in the main text.
